# Intermittent Caloric Restriction Promotes Erythroid Development and Ameliorates Phenylhydrazine-Induced Anemia in Mice

**DOI:** 10.3389/fnut.2022.892435

**Published:** 2022-06-09

**Authors:** Meijuan Bai, Peijuan Cao, Yijun Lin, Pengcheng Yu, Shuo Song, Lingling Chen, Lan Wang, Yan Chen

**Affiliations:** ^1^CAS Key Laboratory of Nutrition, Metabolism and Food Safety, Shanghai Institute of Nutrition and Health, University of Chinese Academy of Sciences, Chinese Academy of Sciences, Shanghai, China; ^2^CAS Key Laboratory of Tissue Microenvironment and Tumor, Shanghai Institute of Nutrition and Health, University of Chinese Academy of Sciences, Chinese Academy of Sciences, Shanghai, China; ^3^School of Life Sciences and Technology, ShanghaiTech University, Shanghai, China

**Keywords:** caloric restriction, erythropoiesis, spleen, bone marrow, anemia

## Abstract

**Background:**

Dietary restriction has a profound effect in altering immune system and promoting metabolic health and aging. However, how dietary restriction impacts erythroid system is largely unknown. We found that a short-term caloric restriction (CR) stimulates expression of KLF1, a master regulator of erythroid development, in the spleen of mouse, and thus explored the potential effect of CR on erythropoiesis.

**Methods:**

We analyzed the effects of intermittent CR and continuous CR for different lengths of time on parameters of peripheral blood and erythroid profiles in the spleen and bone marrow in C57BL/6 mice. We next assessed how different types of CR affect phenylhydrazine-induced anemia in the mice. Colony formation assay was also used to analyze LK + progenitors and BFU-E in the bone marrow.

**Results:**

Intermittent CR for 2 weeks raised the number of reticulocytes in the blood, while continuous CR for 2 weeks elevated red blood cells and hemoglobin level. Intermittent CR for 2 weeks promoted extramedullary hematopoiesis in the spleen, while continuous CR mainly promoted erythropoiesis in the bone marrow. Interestingly, a short-term intermittent CR but not continuous CR was able to ameliorate phenylhydrazine-induced anemia. Intermittent CR reduced early-stage erythroblasts and increased late-stage erythroblasts/mature RBCs in the spleen, indicating an accelerated transition from early-stage to late-stage erythroblasts/mature red blood cells. Furthermore, a short-term intermittent CR elevated LK + progenitors and the committed erythroid progenitor cells BFU-E in the bone marrow.

**Conclusion:**

Our study demonstrated that a short-term intermittent CR, but not continuous CR, has a significant effect to promote hematopoiesis and such activity can ameliorate phenylhydrazine-induced acute anemia in the mouse.

## Introduction

Dietary restriction (DR) or caloric restriction (CR) has been extensively investigated and proved to be effective in prolonging aging and slowing down a plethora of chronic diseases especially metabolic disorders (1, 2). In particular, previous studies have reported beneficial effects of intermittent CR on the treatment of diabetes (3–6), autoimmune encephalomyelitis (7–9), and inflammatory bowel disease (10–12), suggesting a beneficial effect of intermittent CR on immune disorders. A growing body of literature further illustrated the impact of CR on microbiome, innate immune system, adaptive immune system, as well as dynamic migration of immune cells between bone marrow and peripheral blood (13). For example, fasting decreases the number of lymphocytes to about 50% in Peyers’ patches (PPs), while refeeding restores the number of lymphocytes with different composition (14). Naive B cells can migrate from PPs to the bone marrow upon fasting and come back to PPs upon refeeding (14). The short-term fasting also reduces the number of circulating monocytes and improves inflammatory diseases without compromising antimicrobial immunity (15). CR was also found to promote memory T cell accumulation in the bone marrow (16). The memory T cells homing to the bone marrow are associated with enhanced protection against infection and tumor formation (16). However, how CR and especially intermittent CR affects hematopoiesis, especially erythropoiesis, has been largely elusive so far.

Erythroid development is tightly orchestrated by transcription factors among which KLF1, also named EKLF (Erythroid Kruppel-like factor), is a key molecule in defining the erythroid lineage (17). KLF1 is an erythroid-specific transcription factor, which controls erythropoiesis through enhanced expression of genes encoding globin, heme synthesis enzymes, globin chaperones, erythroid cell structural membrane, cytoskeleton proteins, and cell-cycle regulators (18, 19). Recently, KLF1 was found to control cell cycles required for terminal differentiation of erythroblasts before enucleation and maturation into circulating red blood cells (RBCs) (20). As spleen is a prime extramedullary hematopoietic organ in addition to liver in adult, we first analyzed the gene expression profile in the spleen after a short-term CR. Surprisingly, we found that a short-term CR was able to significantly elevate gene expression of *Klf1*. We next carried out a series of studies and uncovered that a short-term intermittent CR was able to affect erythroid development in the spleen and bone marrow. Importantly, we found that a short-term intermittent CR was able to ameliorate PHZ-induced anemia in the mouse.

## Materials and Methods

### Mouse Studies

Six-week-old male wild-type C57BL/6 mice were purchased from Shanghai SLAC Laboratory Animal (Shanghai, China). All animal experimental procedures were authorized by the Institutional Animal Care and Use Committee (IACUC) guidelines of Shanghai Institute of Nutrition and Health, Chinese Academy of Sciences, according to an approval number SINH-2020-CY-1. The mice were maintained in specific pathogen-free environment with constant temperature and humidity and alternating 12-h light/dark cycle. After 1-week adaptation, the mice were divided randomly into two or three groups according to the experimental design. Mice in the control group were fed normal chow *ad libitum*. Mice in the intermittent CR (ICR) group were fed with normal chow at a calorie intake equal to 30% of the free-eating control mice for 3 days, followed by normal chow *ad libitum* for the next 4 days. Mice in the continuous CR (CCR) group were fed with normal chow at 70% calorie of control mice daily. The chow diet was from Pu Lu Teng Biological Technology Co., Ltd. (Shanghai, China).

### Hematological Analysis

A volume of 20 μl of peripheral blood samples from tail tip of mice were collected into EDTA.K_2_ anticoagulant tube (KANG JIAN, Jiangsu, China). Following parameters were analyzed by an automated hematology analyzer (SYSTEM CORPORATION, KOBE, Japan): white blood cell (WBC), RBC, hemoglobin (HGB), hematocrit (HCT), platelet count (PLT), RBC volume distribution width standard deviation (RDW-SD), RBC volume distribution width coefficient of variation (RDW-CV), and reticulocyte (RET).

### Hematoxylin and Eosin Staining of the Spleen

After the mice were sacrificed by asphyxiation, the spleens were taken and fixed overnight with 4% paraformaldehyde. The fixed tissues were sliced into thin slices at 3 μm and stained with Hematoxylin & Eosin (H&E).

### Flow Cytometry

Spleen, femur, and tibia were isolated from the mice. Spleen samples were ground and filtered through a 70 μm filter. Bone marrow samples were obtained by destroying femurs and tibias. All mice samples were incubated in PBS with 2% FBS to keep alive. After centrifugation, the suspended cells in PBS were stained with Fixable Viability Stain 510 live/dead dye (BD Bioscience, San Diego, CA, United States) and mixed cell surface antibodies, respectively, including APC-Cy™7-Rat anti-mouse CD45 (BD Bioscience, San Diego, CA, United States), PE anti-mouse CD71 (Biolegend, San Diego, CA, United States), APC anti-mouse Ter119 (Biolegend), FITC anti-mouse Lineage Cocktail (Biolegend), PE-Cy7 anti-mouse Sca-1 (Biolegend), and Brillant Violet 421 anti-mouse CD117 (Biolegend). Finally, all samples were analyzed by FACS Aria machine (BD Bioscience). Fluorescence-minus-one (FMO) controls were included for all the flow cytometry analyses.

### Quantitative Real-Time PCR

Total RNA of the spleen was separated by Trizol (Invitrogen) according to the manufacturer’s instructions, and then reverse-transcribed into cDNA. The mRNA level was measured by quantitative PCR using SYBR Green master mix (TOYOBO at Shanghai, Shanghai, China). The Cq value of the PCR was used to calculate the relative mRNA level. The sequences of the primers are summarized in Table 1.

**TABLE 1:** Primers used in the study.

	Forward	Reverse
*Actin*	GATCATTGCTCCTCCTGAGC	ACTCCTGCTTGCTGATCCAC
*Ccl24*	ATTCTGTGACCATCCCCTCAT	TGTATGTGCCTCTGAACCCAC
*Chil3*	CTGAATGAAGGAGCCACTGA	AGCCACTGAGCCTTCAACTT
*Dpep1*	GCACAACGACTTGCCTTGG	ATGCGGTGTATCACATCCATC
*Exosc6*	ACGCGGGAGTGGAGATGTA	CGGTCCAACTCTCAGTCTGC
*Hmox1*	AAGCCGAGAATGCTGAGTTCA	GCCGTGTAGATATGGTACAAGGA
*Klf1*	AGACTGTCTTACCCTCCATCAG	GGTCCTCCGATTTCAGACTCAC
*Lcn2*	TGGCCCTGAGTGTCATGTG	CTCTTGTAGCTCATAGATGGTGC
*Ngfr*	TGCCGATGCTCCTATGGCTA	CTGGGCACTCTTCACACACTG
*Nrip3*	TTTTACTCAGGACTCCTCACCG	CTTGGACGAGCCCAGCTTT
*Pde11a*	AACAGGACCTACGATGAACAGG	TGAGGCAGATTCACCCTCGAT
*Pxmp2*	CTCGCCCAATACTTGCTGCT	CAGTGGACCTGTGACAAACAA
*Rnf212*	ATGAGATCATCACAACAACCAGC	TGGAGGAGTCAGGTCAATATCC
*Tac2*	GTGACATGCACGACTTCTTTGT	GGTGTTCTCTTCAACCACGTC
*Trnp1*	TTGGTCTGAGAAATCCCTGC	CGCTGTGTCTATCTGAGGAAG
*Zfp9*	CGTGACATTAAGGGATGTTGCT	CCACACGATAACCCACAGAGA
*Zscan10*	GGCTCAGAGGAATGCGTTAG	CATCTACAGGCCCACCAGTT
*Cd274*	GCTCCAAAGGACTTGTACGTG	TGATCTGAAGGGCAGCATTTC
*Ctla4*	GCTTCCTAGATTACCCCTTCTGC	CGGGCATGGTTCTGGATCA
*Pdcd1*	ACCCTGGTCATTCACTTGGG	CATTTGCTCCCTCTGACACTG
*Gata1*	CAGAACCGGCCTCTCATCC	TAGTGCATTGGGTGCCTGC
*Epor*	GGGCTCCGAAGAACTTCTGTG	ATGACTTTCGTGACTCACCCT
*Fam132b*	ATGGGGCTGGAGAACAGC	TGGCAT-TGTCCAAGAAGACA
*Tfrc*	AAGTCCAGTGTGGGAACAGG	AACCACTCAGTGGCACCAAC

### RNA Sequencing

The RNA quality of the spleen was guaranteed by 2100 Bioanalyzer (Agilent, Santa Clara, CA, United States) and quantified through ND-2000 (Thermo Scientific, United States). RNA-seq transcriptome library was performed according to the instruction of TruSeq™ RNA sample preparation Kit of Illumina (San Diego, CA), and then sequenced by the Illumina HiSeq xten/NovaSeq 6000 sequencer (2 × 150 bp read length). The trimmer and quality control of the raw paired end reads were conducted using SeqPrep^1^ and Sickle^2^ with default parameters. Then, the clean reads were, respectively, aligned to reference genome or transcriptome under orientation mode with the HISAT2 software (21). The mapped reads of every sample were aggregated assembly by StringTie in a reference-based way (22). In detail, the coincident pairs were first assembled into superreads, and then the superreads were compared with the reference genome and the graph of splice sites was constructed. Finally, the paths with high read coverage were retained and assembled together to form transcripts. The clean reads were aligned to reference genome^3^ to obtain all transcripts/genes. Differentially expressed genes and transcripts were analyzed by DESeq2 (23) with TPM as the expression level. Remarkable DEGs and DETs were both determined with *p*-adjust < 0.01 and fold change > 2 or < 0.5. GO and KEGG pathway enrichment were analyzed by Goatools (24). All data analyses were administrated on an online platform of Majorbio Cloud Platform for free^4^ (Shanghai Majorbio Bio-pharm Technology Co., Ltd.). The raw data of the RNAseq have been uploaded to NCBI BioProject Databases (http://www.ncbi.nlm.nih.gov/bioproject/817551).

### Colony-Forming Assay

The LSK^+^ (Lin^–^Sca-1^+^C-kit^+^) cells were collected by FACS from bone marrow of the mice and incubated in PBS containing 2% FBS and 2% P/S. A total of 3,000 LSK^+^ cells were added to each of 35 mm dishes containing 1.2–1.4 ml methylcellulose-based medium with recombinant cytokines (including EPO, SCF, IL-3, and IL-6) (STEMCELL Technologies, Canada). Subsequently, six 35 mm dishes with the LSK^+^ cells were placed into a 15 cm dish (together with a 35 mm dish containing sterile distilled water) and then cultured under 37°C and 5% CO_2_ for 10–12 days. The colonies formed in the 35 mm dishes were analyzed under optical microscope (Olympus Corporation, Japan) and the types of colonies were determined as instructed by MethoCult™ kit (STEMCELL Technologies).

### Statistical Analysis

All data were shown as mean ± S.E.M. The statistical differences between the groups were analyzed by *Student’*s t-test using GraphPad Prism v.7.0. The same software was also used to draw graphical results. The FlowJo-V10 software was used to examine the proportion of erythroid cells.

## Results

### A Short-Term Caloric Restriction Affects Pathways of Erythroid Development and Elevates *Klf1* Expression in the Spleen

**FIGURE 1:**
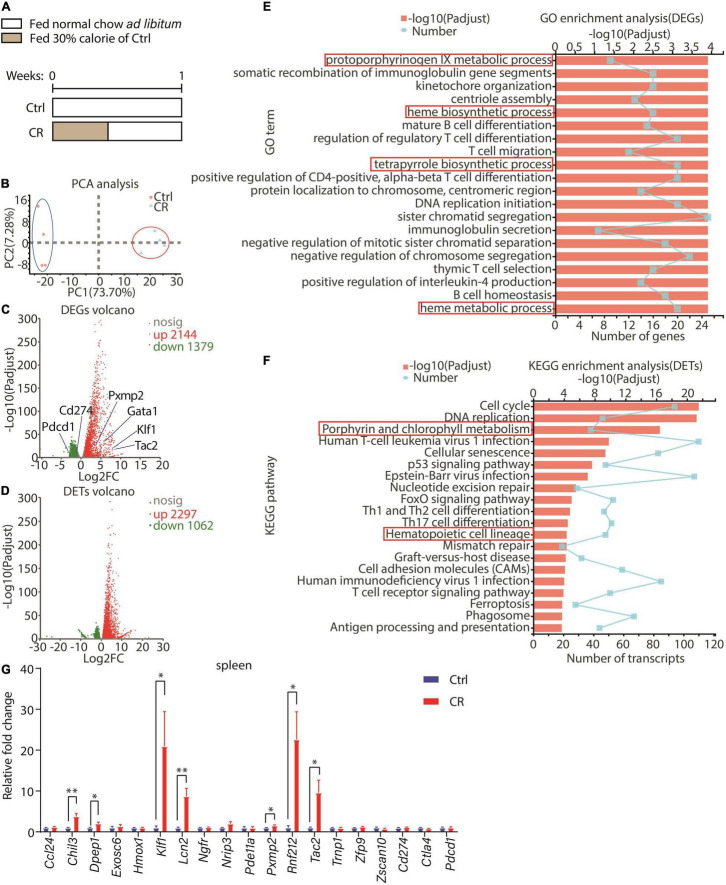
Effect of a short-term CR on gene expression profile in spleen. **(A)** A schematic diagram showing the design of the experiment. Seven-week-old male C57BL/6 mice were divided into two groups: control with normal chow *ad libitum* (n = 4) and CR with 30% calorie intake for 3 days followed by free eating for 4 days (*n* = 4). **(B)** Principal components analysis (PCA) was conducted between control and CR groups according to RNAseq data of the spleen. **(C,D)** Volcanos of differential expressed genes (DEGs) or transcripts (DETs) between the two groups. The data were both filtrated with *P*-adjust < 0.01 and fold change > 2 or < 0.5. **(E)** GO enrichment analysis of DEGs with top 20 GO terms shown here. The data were filtrated with *P*-adjust < 0.5 between the two groups. **(F)** KEGG enrichment analysis of DETs with top 20 KEGG pathways shown here. The data were filtrated with *P*-adjust < 0.5 between the two groups. **(G)** Results of quantitative RT-PCR for representative genes (*n* = 4 for each group). The data are presented as mean ± S.E.M. *P < 0.05 and ^**^P < 0.01.

In an effort to explore the potential functions of CR on immune system, we analyzed the gene expression prolife in the spleen of male C57BL/6J mice after a short-term CR. In the control group, the mice were fed with normal chow *ad libitum*. In the CR group, the mice were fed with 30% calorie of the control group for the first 3 days, followed by 4 days of normal chow *ad libitum* (Figure 1A). The spleens of the mice were used in RNA sequencing (RNAseq). Principal components analysis (PCA) indicated that the overall gene expression profile was significantly different between the two groups (Figure 1B). A total of 3,523 genes were differentially expressed between the two groups with the standard of *P*-adjust < 0.01 and log_2_ fold change > I1.0I, including 2,144 upregulated genes and 1,379 downregulated genes in the CR group (Figure 1C). At the transcript level, 3,359 transcripts were observed to be differentially expressed between the two groups (*P*-adjust < 0.01, log_2_ fold change > I1.0I), including 2,297 upregulated transcripts and 1,062 downregulated transcripts in the CR group (Figure 1D). Next, we performed gene ontology (GO) analysis with differentially expressed genes (DEG) between the two groups. Intriguingly, we found that the gene clusters related to erythroid development, including protoporphyrinogen IX metabolic process, heme biosynthetic and metabolic process, and tetrapyrrole biosynthetic process were altered by CR (Figure 1E). Interrogation of the transcripts using Kyoto Encyclopedia of Genes and Genomes (KEGG) analysis revealed that the transcripts related to porphyrin and chlorophyll metabolism and hematopoietic cell lineage pathways were significantly upregulated in the CR group (Figure 1F). Among the genes significantly upregulated by CR, we found that *Klf1*, a master regulator of erythroid development (20), was upregulated by CR (Figure 1C). Furthermore, upregulation of *Klf1* by CR was confirmed by quantitative RT-PCR in the spleen (Figure 1G). Therefore, the RNAseq results led us to surmise that a short-term CR might regulate erythrocyte development in mice.

### Intermittent Caloric Restriction and Continuous Caloric Restriction Differentially Affect Maturation of Red Blood Cells and Erythropoiesis

**FIGURE 2:**
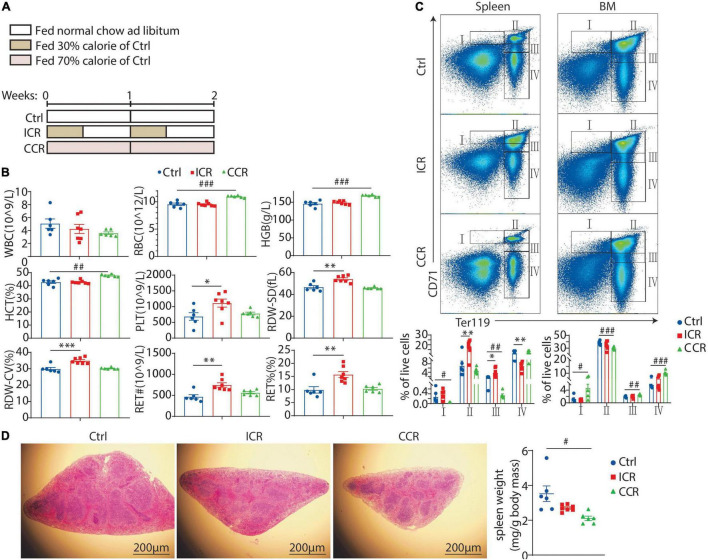
Effect of a short-term CR on erythrocyte development in mice. **(A)** A schematic diagram showing the design of the experiment. Seven-week-old male C57BL/6 mice were divided into three groups (*n* = 6 for each group). **(B)** Blood count of the mice using peripheral blood, including white blood cell (WBC), red blood cell (RBC), hemoglobin (HGB), hematocrit (HCT), platelet count (PLT), red blood cell volume distribution width standard deviation (RDW-SD), red blood cell volume distribution width coefficient of variation (RDW-CV), and reticulocyte (RET). **(C)** The proportion of erythroid cells at different stages in the spleen and bone marrow, including stage I (CD71^hi^Ter119^int^, proerythroblast), stage II (CD71^hi^Ter119^hi^, basophilic erythroblast), stage III (CD71^int^Ter119^hi^, mixture of polychromatophilic erythroblast and orthochromatic erythroblast and reticulocyte), and stage IV (CD71^–^ Ter119^hi^, mature RBCs). **(D)** Representative images of H&E staining of the spleen with its weight shown in the right panel. Scale bars: 200 μm. All the data are presented as mean ± S.E.M. *P < 0.05, ^**^P < 0.01, ^***^P < 0.001 for difference between control and intermittent CR groups. ^#^*P* < 0.05, ^##^ *P* < 0.01, ^###^ *P* < 0.001 for difference between control and continuous CR groups.

We next explored how intermittent CR and continuous CR impacted erythroid development in the mice as the two modes of CR might affect biological processes differently (25). Male C57BL/6J mice were divided into three groups (Figure 2A). The control group was fed with normal chow *ad libitum*. In the intermittent CR (ICR) group, the mice were fed with normal chow at 30% calorie of the control group for 3 days, followed by normal chow *ad libitum* for 4 days each week. The mice in the continuous CR (CCR) group were fed with normal chow at 70% of calorie for the entire period so that the total calorie intake of this group was equivalent to the intermittent CR group. In the first experiment, CR was administered for 2 weeks (Figure 2A). Only the mice in continuous CR group had a significant decrease in body weight (data not shown). We collected peripheral blood from the tail of the mice and analyzed following parameters: WBCs, RBCs, hemoglobin concentration (HGB), hematocrit (HCT), platelets (PLT), red blood cell distribution width (RDW-SD), red blood cell distribution width coefficient of variation (RDW-CV), reticulocyte number (RET#), and reticulocyte percentage (RET%) (Figure 2B). Interestingly, we found that intermittent CR and continuous CR affected these parameters differently. In short, intermittent CR raised PLT, RDW-SD, RDW-CV, RET#, and RET% (Figure 2B). In contrast, continuous CR elevated RBC, HGB, and HCT (Figure 2B).

We next analyzed in detail how CR affected erythroid development. Previous studies have identified five distinct stages of erythrocyte maturation from erythroid progenitors to mature RBCs, namely, proerythroblast, basophilic erythroblast, polychromatophilic erythroblast, orthochromatic erythroblast, and the enucleated reticulocyte, which finally evolves to mature RBC (20). The classical markers used to characterize the process of erythrocyte maturation are CD71 and Ter119. We isolated the cells from spleen and bone marrow of the mice and analyzed the expression of CD71 and Ter119 by FACS. Based on previous studies (26), we identified four populations of cells, namely, stage I (CD71^hi^Ter119^int^, proerythroblast), stage II (CD71^hi^Ter119^hi^, basophilic erythroblast), stage III (CD71^int^Ter119^hi^, mixture of polychromatophilic erythroblast, orthochromatic erythroblast and reticulocyte), and stage IV (CD71^–^ Ter119^hi^, mature RBCs) (Figure 2C). In the spleen, intermittent CR increased the cells at stage II and stage III, but reduced the cells at stage IV (Figure 2C). Continuous CR, on the contrary, reduced cells at stages I and III (Figure 2C). In the bone marrow, intermittent CR had no effect on 4 stages of cells, while continuous CR elevated cells at stage I, III, and IV and reduced the cells at stage II (Figure 2C). Overall, these results suggested that a short-term intermittent CR might promote extramedullary hematopoiesis, while a short-term continuous CR likely might promote erythropoiesis in the bone marrow and inhibit erythropoiesis in the spleen. Of note, we observed that the weight of spleen was significantly reduced by continuous CR in the mice (Figure 2D). However, the overall morphology of the red pulp and white pulp of the spleen was not very different among the three groups by H&E staining (Figure 2D).

**FIGURE 3:**
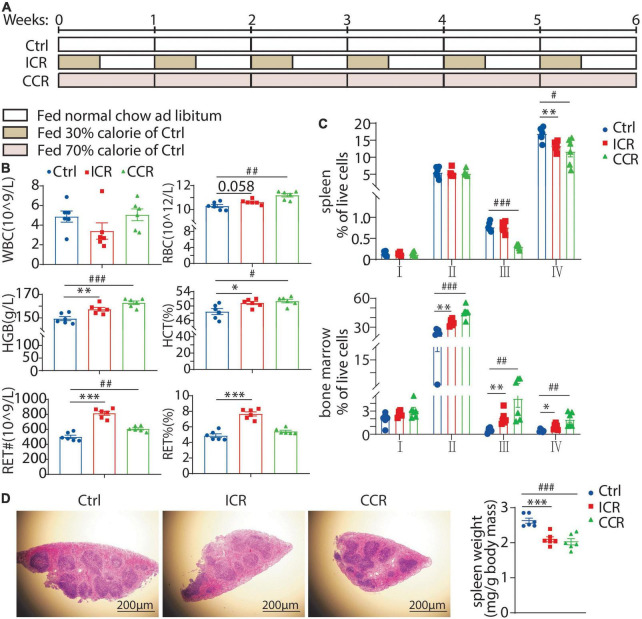
Effect of a long-term CR on erythrocyte development in mice. **(A)** Design of the experiment. Seven-week-old male C57BL/6 mice were divided into three groups (*n* = 6 for each group). **(B)** Blood count of the mice using peripheral blood. **(C)** The proportion of erythroid cells at different stages in the spleen and bone marrow. **(D)** Representative images of H&E staining of the spleen with its weight shown in the right panel. Scale bars: 200 μm. All the data are presented as mean ± S.E.M. *P < 0.05, ^**^P < 0.01, ^***^P < 0.001 for difference between control and intermittent CR groups. ^#^ *P* < 0.05, ^##^ *P* < 0.01, ^###^ *P* < 0.001 for difference between control and continuous CR groups.

We next analyzed how a long-term CR affected erythroid development. The mice were treated with intermittent CR or continuous CR for 6 weeks (Figure 3A). We found that only the mice in continuous CR group had a significant decrease in body weight (data not shown). Both intermittent CR and continuous CR could elevate RBC, HGB, HCT, and RET# (Figure 3B), indicating that both modes of CR can stimulate erythropoiesis in the mice. FACS analysis with CD71 and Ter119 antibodies revealed that intermittent CR reduced cells at stage IV and continuous CR reduced cells at stages III and IV in the spleen (Figure 3C), indicating a likely reduced extramedullary hematopoiesis in the spleen. This notion was supported by the finding that both types of the CR significantly reduced the weight of the spleen (Figure 3D). On the contrary, both intermittent CR and continuous CR elevated the cells at stages II, III, and IV in the bone marrow (Figure 3C), indicating an activated erythropoiesis in the bone marrow by both types of CR.

### A Short-Term Intermittent Caloric Restriction Ameliorates Acute Hemolytic Anemia Induced by Phenylhydrazine

**FIGURE 4:**
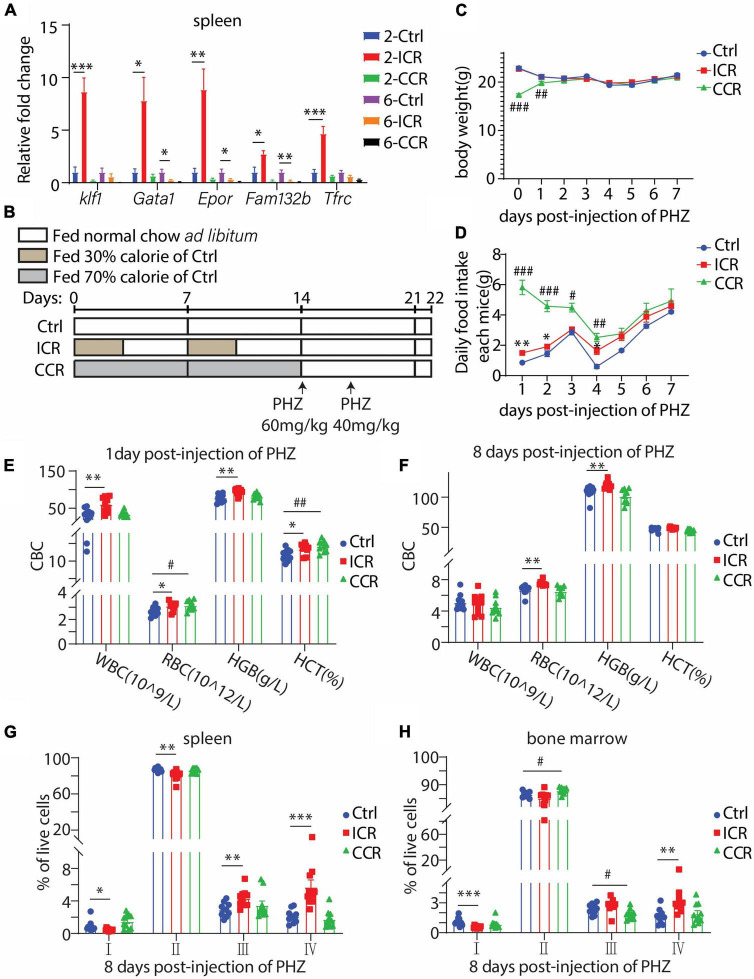
Effect of CR on acute hemolytic anemia induced by PHZ in mice. **(A)** Gene expression levels of crucial genes involved in erythrocyte development in the spleen under a short-term or long-term CR for Figures 2, 3 (*n* = 6 for each group). **(B)** Design of the experiment. Seven-week-old male C57BL/6 mice were divided into three groups (*n* = 10 for each group). The mice were *i.p.* injected with 60 mg/kg and 40 mg/kg PHZ, respectively, on the first and third days after the end of CR. All mice were fed normal chow *ad libitum* since receiving PHZ. **(C,D)** Body weight and food intake of the mice post-injection of PHZ. **(E,F)** Blood count of the mice using peripheral blood. **(G,H)** The proportion of erythroid cells at different stages in the spleen and bone marrow. All the data are presented as mean ± S.E.M. *P < 0.05, ^**^P < 0.01, ^***^P < 0.001 for difference between control and intermittent CR groups. ^#^ *P* < 0.05, ^##^ *P* < 0.01, ^###^ *P* < 0.001 for difference between control and continuous CR groups.

In the RNAseq result as shown in Figure 1, we found that *Klf1* expression was significantly elevated by a short-term CR in the spleen. We further analyzed the spleen samples in mice treated with different types of CR for different times as shown in Figures 2, 3. We assessed the mRNA levels of several key transcription factors and genes involved in erythropoiesis, including *Klf1*, *Gata1, Epor, Fam132b*, and *Tfrc*. *Gata1* is a widely studied erythroid transcription factor whose inactivation in embryos was lethal due to anemia (27). *Epor* encodes erythropoietin receptor that mediates stimulation of proliferation and differentiation of erythroid cells by EPO (28–30). The erythroblast-derived hormone erythroferrone (ERFE), encoded by *Fam132b*, is a secreted protein that specifically inhibits hepcidin production to promote proper iron supply for normal erythropoiesis (31). Transferrin receptor 1 encoded by *Tfrc* is imperative for erythroid development (32–34). Interestingly, we found that the mRNA levels of all the five crucial genes in the spleen were profoundly upregulated by 2 weeks of intermittent CR, but not by other three paradigms of CR (Figure 4A). This result thus indicated that a short-term intermittent CR is likely able to promote erythroid development in the spleen of the mice.

Based on such finding, we analyzed the effect of a short-term CR on acute hemolytic anemia induced by phenylhydrazine (PHZ). The mice were subjected to intermittent CR or continuous CR for 2 weeks, followed by *i.p.* injection of PHZ on the first and third days after the end of CR (Figure 4B). The mice had free access to normal chow since receiving PHZ. As expected, the bodyweight of the mice in the continuous CR group had a significant gain due to an increase in food intake (Figures 4C,D). During the course of PHZ-induced hemolytic anemia, we collected peripheral blood from tail vein on the first and eighth day since injection of PHZ. On the first day post-injection of PHZ, intermittent CR elevated WBC, RBC, HGB, and HCT, while continuous CR increased RBC and HCT (Figure 4E). At the eighth day post-injection of PHZ, intermittent CR group had a significant increase in RBC and HGB, while continuous CR group had no difference compared with the control group (Figure 4F). These results, therefore, indicated that intermittent CR but not continuous CR is able to ameliorate PHZ-induced anemia. Such activity of intermittent CR was further evidenced by analyzing different stages of erythroid development using the spleen cells at the eighth day post-injection of PHZ. At this time point, the cells at stages I and II were reduced by intermittent CR, together with increases in cells at stages III and IV (Figure 4G), indicating an accelerated transition from early-stage erythroblasts to late-stage erythroblasts and mature RBCs. In contrast, the effects of both types of CR on bone marrow erythropoiesis were different. Intermittent CR had a decrease in stage I-cells with an increase in stage IV-cells, and continuous CR had an increase in stage II-cells with a reduction of stage III-cells (Figure 4H).

### A Short-Term Intermittent Caloric Restriction Elevates LK^+^ Progenitors and Committed Erythroid Progenitor Cells BFU-E in Bone Marrow

**FIGURE 5:**
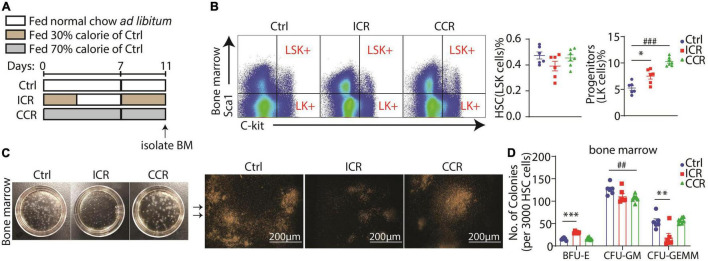
A short-term CR affects hematopoiesis in bone marrow from progenitors. **(A)** Design of the experiment. Seven-week-old male C57BL/6 mice were divided into three groups (*n* = 6 for control group, *n* = 6 for intermittent CR group and *n* = 7 for continuous CR group). **(B)** Proportion of LSK^+^ cells and LK^+^ cells in bone marrow. Quantitation of the FACS results is shown in the right panel. **(C)** Representative images of colonies formed from LSK^+^ cells of the bone marrow. The amplified images are shown in the right panel. Scale bar 200 μm. **(D)** Colony counts of BFU-E, CFU-GM, and CFU-GEMM derived from LSK^+^ cells in the bone marrow. All the data are presented as mean ± S.E.M. *P < 0.05, ^**^P < 0.01, ^***^P < 0.001 for difference between control and intermittent CR groups. ^##^ *P* < 0.01, ^###^ *P* < 0.001 for difference between control and continuous CR groups.

Hematopoiesis from hematopoietic stem cell (HSC) to RBC has been well characterized to follow the pathway HSC > MPP (multipotential progenitor cells) > CMP (common myeloid progenitor) > MEP (megakaryocyte-erythroid progenitor) > BFU-E (burse-forming unit-erythroid) > CFU-e (colony-forming unit-erythroid) > ProE (proerythroblast) > erythroblast > erythrocyte (20). It is well known that GATA1 started its expression in CMPs and reached its highest level when differentiated into ProE in committed erythroid progenitor cells (35). We found a remarkable increase in the mRNA level of *Gata1* in the spleen after two cycles of intermittent CR in Figure 4A, implying that a short-term intermittent CR could potentially affect hematopoiesis from HSC to erythroblasts. We thus analyzed how different types of a short-term CR affected hematopoiesis in the bone marrow (Figure 5A). In particular, we analyzed the proportions of LSK^+^ cells (HSC/MPP, Lin^–^Sca1^+^C-kit^+^) and LK^+^ cells (progenitors, Lin^–^Sca1^–^C-kit^+^) in the bone marrow of the mice by FACS. Interestingly, we found that both intermittent CR and continuous CR could significantly elevate the number of LK^+^ cells, without a change in the LSK^+^ cells (Figure 5B). We next purified LSK^+^ cells and seeded them into methylcellulose-containing growth factors, including EPO to assess the hematopoietic activity of HSC/MPP. Different colonies were formed in the semisolid medium among three mouse groups (Figure 5C). On the one hand, based on the morphology of the colony, we found that a short-term intermittent CR significantly increased BFU-E, together with a significant reduction in CFU-GEMM (colony-forming unit-granulocyte, erythroid, macrophage, megakaryocyte) (Figure 5D). On the other hand, continuous CR decreased CFU-GM (colony-forming unit-granulocyte, macrophage) without affecting BFU-E(Figure 5D). Collectively, these data indicated that intermittent CR but not continuous CR can promote differentiation of HSC/MPP into committed erythroid progenitors, consistent with our finding that intermittent CR but not continuous CR significantly ameliorated PHZ-induced anemia as shown in Figure 4.

## Discussion

In this study, we demonstrated that a short-term intermittent CR, but not continuous CR, has a significant effect to promote extramedullary hematopoiesis in the spleen and such activity can ameliorate PHZ-induced acute anemia in the mouse. Intermittent CR for 2 weeks could strongly stimulate the expression of a set of genes critical for erythropoiesis in the spleen, including *Klf1*, *Gata1, Epor, Fam132b*, and *Tfrc*. Such activity of intermittent CR on erythropoiesis in the spleen was reflected by elevation of reticulocytes in peripheral blood and increases in stage II and stage III erythroblasts (Figure 2C). A short-term intermittent CR also increased the number of BFU-E, the committed erythroid progenitors. Most importantly, a short-term intermittent CR was able to improve PHZ-induced anemia. Intermittent CR resulted in a significant increase in RBC and HGB in PHZ-injected mice. At the eighth day post-injection of PHZ, the cells at stages I and II were reduced by intermittent CR, together with increases in cells at stages III and IV, thus indicating an accelerated transition from early-stage erythroblasts to late-stage erythroblasts and mature RBCs. Collectively, these results suggested that intermittent CR, but not continuous CR, has a prominent effect on extramedullary erythropoiesis in the spleen, in addition to its potential effect on erythropoiesis in the bone marrow.

Although the effects of different types of CR including intermittent CR have been extensively investigated in many physiological and pathophysiological conditions, how CR affects RBC development has been elusive. Early studies revealed that continuous CR at 40% calorie reduction for 8 weeks can shorten the half-life of RBCs without causing anemia in rats (36). In addition, continuous CR by 33% or 66% for 14 days led to decreases in peripheral blood reticulocytes and bone marrow erythroid cells in rats (37). In our study, we found that continuous CR by 70% for 2 weeks elevated RBC, HGB, and HCT, while it reduced cells at stage I and III in the spleen together with increases of cells at stage I, III, and IV in the bone marrow. In addition, continuous CR for 6 weeks reduced cells at stages III and IV in the spleen, while elevated the cells at stages II, III, and IV in the bone marrow. Our results thus indicated that continuous CR has a certain effect to activate erythropoiesis in the bone marrow in mice, different from the result in rat (37). Such discrepancy is likely caused by the differences in animal species and degree of CR. Nevertheless, how intermittent CR affects erythroid development has not been investigated before. Our study clearly indicated that intermittent CR has a unique role in modulating erythropoiesis distinctive from continuous CR. In addition, it is currently unclear why intermittent CR is different from continuous CR in terms of stimulation of erythropoiesis. One possibility is that continuous CR might send a stronger starvation signal to the body than intermittent CR. Such a starvation signal may guide the body to preserve tissue iron concentrations. For example, fasting has been shown to raise blood level of hepcidin-25 in healthy individuals and presumed to reduce iron absorption under fasting condition (38). The short-term fasting was also proposed to bring about numerous health benefits rapidly upon the initiation of CR, such as more efficient fuel usage to adapt to food deprivation, upregulation of survival signaling to avoid cell death, and increase of gene expression involved in cytoprotection (39). However, these benefits might fade during a long-term continuous CR.

Our studies also revealed that a short-term CR was able to elevate LK^+^ progenitors and committed erythroid progenitor cells BFU-E in bone marrow. Previously, it was found that prolonged fasting has a pro-regenerative effect on HSC through decreasing the level of IGF-1 or PKA (40). In addition, adult-onset DR could ameliorate the aging phenotypes of HSCs, increase HSC quiescence, and improve HSC repopulation at the expense of limiting the capacity of lymphoid differentiation of HSC (41). It is thus important to be determined in the future whether a short-term CR is different from prolonged CR in their capacity to alter the development of various lineages of HSCs.

## Conclusion

Our study demonstrated that a short-term intermittent CR, but not continuous CR, has a significant effect to promote hematopoiesis. In addition, a short-term intermittent CR is able to ameliorate phenylhydrazine-induced acute anemia in the mouse. These results thus revealed a unique role of CR on erythropoiesis in the body.

## Data Availability Statement

The raw data supporting the conclusions of this article will be made available by the authors, without undue reservation.

## Ethics Statement

The animal study was reviewed and approved by Institutional Animal Care and Use Committee (IACUC), Shanghai Institute of Nutrition and Health, Chinese Academy of Sciences.

## Author Contributions

YC and MB conceptualized and designed the study. MB performed the experiment. PC, YL, PY, SS, LC, and LW provided the technical assistance. MB and YC wrote the manuscript and prepared the figures. All authors read and approved the manuscript.

## Conflict of Interest

The authors declare that the research was conducted in the absence of any commercial or financial relationships that could be constructed as a potential conflict of interest.

## Publisher’s Note

All claims expressed in this article are solely those of the authors and do not necessarily represent those of their affiliated organizations, or those of the publisher, the editors and the reviewers. Any product that may be evaluated in this article, or claim that may be made by its manufacturer, is not guaranteed or endorsed by the publisher.

## Footnotes

1
https://github.com/jstjohn/SeqPrep

2
https://github.com/najoshi/sickle

3
http://asia.ensembl.org/Mus_musculus/Info/Index

4
www.majorbio.com

## Abbreviations

BFU-E: burse-forming unit-erythroid
CFU-e: colony-forming unit-erythroid
CMP: common myeloid progenitor
CR: caloric restriction
DEG: differentially expressed genes
DR: dietary restriction
GO: gene ontology
HCT: hematocrit
HGB: hemoglobin
KEGG: Kyoto Encyclopedia of Genes and Genomes
MEP: megakaryocyte-erythroid progenitor
MPP: multipotential progenitor cells
PCA: Principal components analysis
PHZ: phenylhydrazine
PLT: platelet count
PP: Peyers’ patch
ProE: proerythroblast
RBC: red blood cell
RDW-CV: red blood cell volume distribution width coefficient of variation
RDW-SD: red blood cell volume distribution width standard deviation
RET: reticulocyte
RNAseq: RNA sequencing
WBC: white blood cell.
